# Willingness to Participate in Longitudinal Research Among People with Chronic Pain Who Take Medical Cannabis: A Cross-Sectional Survey

**DOI:** 10.1089/can.2017.0051

**Published:** 2018-03-01

**Authors:** Marcus A. Bachhuber, Julia H. Arnsten, Joanna L. Starrels, Chinazo O. Cunningham

**Affiliations:** Division of General Internal Medicine, Department of Medicine, Albert Einstein College of Medicine/Montefiore Medical Center, Bronx, New York.

**Keywords:** medical marijuana, marijuana, chronic pain, cohort studies, analgesics

## Abstract

**Background:** Regulatory barriers limit clinical trials of medical cannabis in the United States. Longitudinal cohort studies may be one feasible alternative that could yield clinically relevant information. Willingness to participate in such studies is not known.

**Materials and Methods:** In October 2016, we surveyed a convenience sample of patients with chronic pain from two New York registered organizations (responsible for growing, processing, distributing, and retailing medical cannabis products). After a vignette describing a longitudinal cohort study involving weekly patient-reported outcomes and quarterly assessments of physical functioning and urine and blood tests, we asked about respondents' willingness to participate. We examined willingness to participate, duration of participation, and frequency of data collections overall and by subgroups, using multivariable logistic regression models.

**Results:** Of 405 respondents (estimated response rate: 30%), 54% were women and 81% were white non-Hispanic. Neuropathy was the most common pain condition (67%) followed by inflammatory bowel disease (19%). Of respondents, 94% (95% CI 92–97%) thought that the study should be done, 85% (95% CI 81–88%) would definitely or probably enroll if asked, 76% (95% CI 72–81%) would participate for ≥1 year, and 59% (95% CI 54–64%) would respond to questions at least daily. Older age was the only factor associated with lower willingness to participate, lower willingness to participate for ≥1 year, and lower willingness to respond to questions at least daily.

**Conclusions:** Nearly all respondents were supportive of the proposed study and most reported that they would enroll if asked. Enhanced engagement with older individuals may be needed to promote equal enrollment. Recruitment for longitudinal cohort studies with frequent data collection appears feasible in this patient population.

## Introduction

Chronic pain is common in the United States, and its management is challenging. Over the past two decades, opioid analgesics have become a leading pain management strategy and dispensing has tripled.^1^ In parallel, the incidence of opioid use disorder and opioid overdoses have both dramatically increased.^1,2^ To reduce these harms, patient groups, clinicians, and policymakers have called for new strategies to address pain management and reduce use of opioid analgesics. One important and rapidly expanding strategy to manage chronic pain is the use of medical cannabis. As of January 2018, medical use of cannabis is legal in 29 states and the District of Columbia.

In January 2017, the National Academies of Science, Engineering, and Medicine released a landmark report finding substantial evidence for cannabis' efficacy in treating chronic pain.^3^ But beyond evidence of efficacy, there are numerous gaps in research, including patient selection, long-term treatment outcomes, and dosing of major cannabinoids such as Δ9-tetrahydrocannabinol (THC) and cannabidiol (CBD). While randomized controlled trials (RCTs) are the “gold standard” of evidence, RCTs of medical cannabis (a Schedule I substance) currently face numerous hurdles in the United States both due to product availability and due to restrictive dispensing and security procedures required. Although the Drug Enforcement Administration announced a policy change to expand the number of cannabis manufacturers, currently only one entity is authorized to produce and supply cannabis to U.S. researchers.^3,4^ The cannabis products available for research are limited in scope and not necessarily comparable to cannabis products available in state dispensaries.^3^ Even when products are obtained for research, they typically must be dispensed in a directly observed setting. Because of these restrictions, over the last 41 years, only six clinical trials in the United States have administered cannabis to examine its effect on pain. These randomized trials all occurred in tightly controlled settings (human laboratories), for short durations (4 h to 12 days), with small sample sizes (10–55 participants).^5–10^

Randomized trials might also face several other challenges. Unlike a novel pharmaceutical, many people with chronic pain have used cannabis and may bring those experiences to a study, potentially influencing results. However, in contrast, enrolling participants who are cannabis-naive may not necessarily be representative of the broader patient population because of such widespread use. In addition, strong political views and a desire to advocate for medical cannabis access may lead to enrollment of participants who are invested in a certain outcome. Finally, people with chronic pain may not be motivated to enroll in a clinical trial because of cannabis' widespread availability outside of the trial.

Given current limitations of interventional research, observational studies are an appealing alternative. Longitudinal cohort studies of patient-reported pain outcomes are feasible, and even intensive assessments of pain (i.e., several times daily) have not been found to affect participants' responses.^11,12^ While longitudinal cohort studies that simply compare those who use medical cannabis to those who do not would be inescapably confounded, more complex designs and analyses could potentially come closer to estimating causation.^13^ From a clinical perspective, studying medical cannabis in a naturalistic setting may also be more aligned with how the system currently operates. Physicians do not prescribe medical cannabis such as other medications, but they certify patients to purchase it. Patients then choose the dose, amount, and route of administration.

Despite the urgent need for research on medical cannabis and chronic pain, willingness of the target population to participate in such research is not known. Frustration with previous treatment failures and a desire for new options may motivate patients to participate in this line of research. In contrast, experiences of stigma and marginalization from the healthcare system may dissuade patients from participating.^14,15^ The purpose of the current study is to examine willingness to participate in research among patients with chronic pain who take medical cannabis. Overall and by demographic (i.e., age and sex) and clinically important subgroups (e.g., taking opioid analgesics), we describe willingness to participate in a longitudinal study, duration for which potential participants would join in the study, and frequency with which potential participants would provide data.

## Materials and Methods

### Setting and population

The setting for the current study is New York State. New York State's medical cannabis program (operational in January 2016) has several features that make it a promising venue for longitudinal research on medical cannabis and chronic pain. To be certified for medical cannabis, individuals must have at least one qualifying condition (cancer, neuropathy, HIV/AIDS, inflammatory bowel disease, spinal cord injury, multiple sclerosis, epilepsy, and several other neurological diseases) and one associated complication (severe or chronic pain, cachexia or wasting syndrome, severe nausea, seizures, or severe or persistent muscle spasms). After a program expansion in March 2017, after this study was conducted, patients with severe or chronic pain from any condition qualify (not just the conditions listed above). Patients can only purchase medical cannabis products from state-licensed dispensaries, which offer products with a variety of THC:CBD ratios with several different routes of administration (sublingual tincture, PO oil or capsule, and oil for vaporization)—whole plant material is not available in New York dispensaries. As all products are third-party tested for content and allow for dosing in milligrams, cannabis use can be measured with relative precision.

We surveyed customers of two New York State registered organizations (responsible for growing, processing, distributing, and retailing medical cannabis products). In October 2016, we recruited a convenience sample of patients with chronic pain by posting fliers in dispensaries, approaching patients in waiting rooms, and sending out an electronic link via patient newsletters. Recruitment materials specified that the survey was for patients with chronic pain, but did not specify that the topic would be willingness to participate in clinical research. Respondents were offered a $15 gift card for completion of the survey. Inclusion criteria were as follows: (1) age ≥18 years, (2) registration as a patient with the New York State medical cannabis program, (3) chronic or severe pain as a qualifying complication (by self-report), and (4) able to comprehend English. We excluded respondents who completed the survey in less than 90 sec. While we could not determine the exact number of eligible patients, we estimated that the two registered organizations served 30% of an estimated 6000 medical cannabis patients in New York at the time of the survey, 75% of which have chronic or severe pain,^16^ for a total estimate of 1350 eligible patients.

### Survey

We created a 28-question survey to examine willingness to participate in longitudinal cohort studies among patients with chronic pain who take medical cannabis. Based on potential research participants' information needs as described in previous work, we created a vignette describing a hypothetical longitudinal cohort study involving patient-reported outcomes as well as assessments of physical functioning and urine and blood tests (Supplementary Data).^17,18^

After reading the description, we first asked respondents, “Do you think this study should be done?” (answer choices: definitely yes, probably yes, probably no, and definitely no). We then asked respondents' willingness to participate through a series of three questions: (1) “Would you participate in the study if you were asked?” (answer choices: definitely yes, probably yes, probably no, and definitely no), (2) “If you joined the study, would you be willing to participate for”: (answer choices: 5 years, 2 years, 1 year, 6 months, 3 months, and I would not join the study), and (3) “If you were texted or prompted on your cell phone to answer questions from the study, how often would you be willing to respond?” (answer choices: twice or more daily, once daily, three times a week, once a week, twice a month, once a month, less than once a month, and I would not join the study). We drew the wordings and based our answer choices for these questions from a recent study.^19^

For respondents' indicating a willingness to participate in the proposed study (for any length of time), we presented a list of potential reasons why and asked participants to check all that apply. Similarly, for respondents not willing to participate, we displayed a list of potential reasons why not and asked respondents' to check all that apply. We drew these potential reasons from studies examining willingness to participate in diverse types of research.^19–38^

In addition to collecting information on respondents' willingness to participate, we collected information about respondents' sociodemographic characteristics, pain condition and medication use, and information about medical cannabis qualifying conditions and product use. For sociodemographic characteristics, we collected age, sex, race/ethnicity (Asian/pacific islander, black non-Hispanic, white non-Hispanic, Hispanic/Latino of any race, and any other race or multiple races), education level, income, employment, and health insurance. For pain condition, we collected information on the duration of the pain condition and the frequency of pain, as well as a three-item measure of pain intensity and interference with functioning (the PEG scale).^39^ We classified pain and interference scores from the PEG (mean of the three items) into mild, moderate, and severe using established cutoffs.^40^ For pain medication use, we asked about use of common medication types in the past 30 days, and for respondents reporting opioid analgesic use, we asked about the number of days used in the past 30 days. For medical cannabis information, we collected the number of months the respondent has used medical cannabis, number of days in the past 30 days used, dosage forms (high THC, balanced THC:CBD, high CBD), and routes of administration (sublingual, oral, vapor).

### Missing data

Of all respondents, 83% (*n*=338/405) provided complete data. To account for missing data, we conducted a multiple imputation procedure with chained equations.^41^ We performed this in stages. First, we imputed sociodemographic variables, followed by pain condition and medication use variables, then use of New York State medical cannabis products, and finally, willingness to participate in research. We assumed data were missing at random and created 20 imputed datasets. All data presented include 95% confidence intervals (95% CI) to account for the uncertainty around the imputed data.

### Statistical analysis

First, we calculated descriptive statistics (mean, median, frequencies) along with 95% CIs for all survey questions. Next, to describe willingness to participate in research among specific subgroups, we used multivariable logistic regression models. Our main outcomes were the three questions on willingness to participate, and we dichotomized responses for these questions. For the question, “Would you participate in the study if you were asked?,” we dichotomized responses as “definitely yes” and “probably yes” compared to “probably no” and “definitely no.” For the question, “If you joined the study, would you be willing to participate for?,” we dichotomized responses as “≥1 year” and “<1 year or would not participate.” For the question, “If you were texted or prompted on your cell phone to answer questions from the study how often would you be willing to respond?,” we dichotomized responses as “once or more daily” and “less than daily.” We selected independent variables for our models based on factors previously found to be associated with willingness to participate (i.e., age, sex, race/ethnicity, education) and variables that delineate clinically important subgroups (i.e., cancer versus noncancer pain, severe versus mild/moderate pain, interference with function, and use of opioid analgesics in the previous 30 days).^18–21,23,24,29–37,42–46^ To avoid small cell sizes, we collapsed levels of these categorical variables into clinically meaningful dichotomies. For each model, we estimated the predicted probability of the outcome (e.g., the percentage of women who would participate in the study if asked) using predictive margins. We also estimated contrasts (e.g., the difference in the percentage of women who would participate compared with men). Analyses were conducted using SAS 9.4 and Stata 13. This research was approved by the Albert Einstein College of Medicine and Montefiore Medical Center Institutional Review Board (protocol 2016-6728).

## Results

Of 599 respondents, 191 (32%) were excluded due to ineligibility (150 were not registered as patients with New York State, 37 did not report chronic pain as a symptom, and 4 did not consent), and 3 (0.5%) were excluded for taking <90 sec to complete the survey. Based on our estimates of the number of eligible patients, this represents an estimated response rate of 30% (*n*=405/1350).

Sociodemographic characteristics of the final sample are shown in Table 1. The majority were women (54%, 95% CI 49–59%), white non-Hispanic (81%, 95% CI 77–85%), and had college or graduate degrees (35%, 95% CI 30–40% and 25%, 95% CI 20–29%, respectively).

**Table 1.** **Demographic and Clinical Characteristics of New York State Medical Cannabis Patients with Severe or Chronic Pain (*N*=405)**

Characteristic	% (95% CI)
Age, median (*n*=404)	53 (51–55)
Female gender (*n*=405)	54 (49–59)
Race/ethnicity (*n*=404)	
Asian/Pacific Islander	1 (0–2)
Black	5 (3–7)
Hispanic/Latino, of any race	9 (6–12)
Native American/Alaskan Native	1 (0.02–2)
White	81 (77–85)
Other/multiple races	4 (2–6)
Education (*n*=352)	
Less than high school	1 (0.02–2)
High school diploma or GED	12 (8–15)
Some college	27 (23–32)
College degree	35 (30–40)
Graduate degree	25 (20–29)
Income (*n*=342)	
Less than $20,000	20 (16–24)
$20,000–$39,000	15 (12–19)
$40,000–$59,000	15 (11–19)
$60,000–$79,000	11 (7–14)
$80,000 or higher	39 (34–44)
Work status (*n*=354)	
Full time	24 (20–28)
Part time	7 (4–9)
Retired	17 (12–20)
Unemployed	7 (4–10)
Disabled	46 (41–51)
Health insurance (*n*=353)	
Public (Medicare or Medicaid)	47 (43–53)
Private	50 (45–56)
No insurance	2 (1–4)

Not all respondents provided complete information, number of respondents with nonmissing values noted for each item. Data shown reflect data for all respondents after multiple imputation of missing data. Percentages may not add to 100 due to rounding.

GED, General Educational Development certificate.

Neuropathy was the most common pain condition (67%, 95% CI 63–72%) followed by inflammatory bowel disease (19%, 95% CI 15–22%; Table 2). Most respondents reported constant pain (80%, 95% CI 76–84%) and had scores indicating severe pain intensity and interference (56%, 95% CI 51–61%). Of all respondents, 39% (95% CI 34–44%) reported opioid analgesic use.

**Table 2.** **Pain Condition and Duration, Pain Levels, and Medication Use Among New York State Medical Cannabis Patients with Severe or Chronic Pain (*N*=405)**

Characteristic	% (95% CI)
Qualifying conditions (*n*=405)^a^	
Neuropathy	67 (63–72)
Inflammatory bowel disease	19 (15–22)
Spinal cord injury	18 (14–22)
Cancer	15 (12–19)
HIV/AIDS	3 (1–5)
Multiple sclerosis	9 (6–12)
Amyotrophic lateral sclerosis	0.2 (0–0.7)
Parkinson's disease	1 (0.3–3)
How often do you experience pain? (*n*=393)	
It's constant, always there	80 (76–84)
At least once a day	16 (12–19)
At least once a week	3 (2–5)
Not every week, but at least once a month	1 (0.02–2)
Duration of pain condition, years, mean (*n*=390)	10.8 (9.8–11.7)
Pain and interference (PEG) score (*n*=388)	
Mild (0–3)	9 (6–12)
Moderate (4–6)	35 (30–40)
Severe (7–10)	56 (51–61)
Pain medication use in the past 30 days (*n*=381)	
Opioid analgesics	39 (34–44)
Acetaminophen	17 (13–21)
Nonsteroidal anti-inflammatory drugs	37 (32–42)
Tricyclic antidepressants	4 (2–6)
Pregabalin	8 (5–11)
Gabapentin	17 (13–21)
Duloxetine	9 (6–12)
Prescription patch or cream	18 (14–22)
Number of medication types	
0	29 (24–33)
1–2	50 (45–55)
3+	21 (17–25)
Days of opioid analgesic use in past month among those reporting any use, mean	24 (22–26)

Not all respondents provided complete information, number of respondents with nonmissing values noted for each item. Data shown reflect data for all respondents after multiple imputation of missing data. Percentages may not add to 100 due to rounding.

^a^ Respondents may report more than one condition

For medical cannabis, high THC (59%, 95% CI 54–64%) and balanced THC:CBD forms (54%, 95% CI 49–60%, respectively) were used roughly equally, and sublingual tincture was the most common route of administration (57%, 95% CI 52–63%; Table 3). Nearly half of respondents reported using more than one form (44%, 95% CI 39–50%) and more than one route of administration (46%, 95% CI 41–51%).

**Table 3.** **Medical Cannabis Use Among New York State Medical Cannabis Patients with Severe or Chronic Pain (*N*=405)**

Characteristic	% (95% CI)
Duration of medical cannabis use in months, mean (*n*=352)	5.1 (4.7–5.4)
Number of days of medical cannabis use in past month, mean (*n*=359)	21 (19–22)
Dosage form (*n*=370)	
High THC	59 (54–64)
Balanced THC:CBD	54 (49–60)
High CBD	37 (32–42)
More than one dosage form	44 (39–50)
Route of administration (*n*=370)	
Sublingual tincture	57 (52–63)
Oral capsule or oil	29 (24–34)
Vapor oil	64 (59–69)
More than one route of administration	46 (41–51)

Not all respondents provided complete information, number of respondents with nonmissing values is noted for each item. Data shown reflect data for all respondents after multiple imputation of missing data. Percentages may not add to 100 due to rounding.

CBD, cannabidiol; THC, tetrahydrocannabinol.

Nearly all respondents thought that the study described should be done (94%, 95% CI 92–97%), and the vast majority reported that they would enroll if asked (85%, 95% CI 81–88% definitely or probably yes; Table 4). Almost three-quarters would participate for 1 year or longer (76%, 72–81%), and over half would respond to questions at least daily (59%, 95% CI 54–64%). Increasing age was significantly associated with a lower willingness to participate (−3 percentage points per decade, 95% CI −5 to −0.3), lower willingness to participate for ≥1 year (−3 percentage points per decade, 95% CI −6 to −0.2), and lower willingness to respond to questions at least daily (−5 percentage points per decade, 95% CI −8 to −1; Table 4). Women were more likely than men to report being willing to respond to questions at least daily (12 percentage points, 95% CI 2–22). There were no significant differences in willingness to participate by race/ethnicity, education level, cancer status, pain and interference score, or use of opioid analgesics.

**Table 4.** **Willingness to Participate in a Longitudinal Cohort Study Among New York State Medical Cannabis Patients with Severe or Chronic Pain**

	Willing to participate		Would participate for ≥1 year		Would respond to questions by phone at least daily	
Characteristic	% (95% CI)	Adjusted difference,% (95% CI)	% (95% CI)	Adjusted difference,% (95% CI)	% (95% CI)	Adjusted difference,% (95% CI)
Overall	85 (81 to 88)		76 (72 to 81)		59 (54 to 64)	
Age		−3 (−5 to −0.3)^a,*^		−3 (−6 to −0.2)^*^		−5 (−8 to −1)^**^
30	91 (86 to 96)		83 (76 to 90)		69 (61 to 78)	
45	87 (83 to 91)		79 (74 to 84)		62 (57 to 68)	
65	81 (75 to 86)		72 (65 to 78)		52 (45 to 60)	
Sex						
Male	84 (79 to 90)	Ref.	75 (69 to 82)	Ref.	53 (45 to 60)	Ref.
Female	85 (79 to 90)	1 (−6 to 8)	77 (71 to 83)	2 (−7 to 11)	65 (58 to 71)	12 (2 to 22)^*^
Race/ethnicity						
White, non-Hispanic	87 (79 to 96)	Ref.	73 (63 to 84)	Ref.	55 (43 to 67)	Ref.
Any other race/ethnicity	84 (80 to 88)	−3 (−13 to 7)	77 (72 to 82)	4 (−8 to 15)	60 (55 to 65)	5 (−9 to 18)
Education						
College degree or more	84 (80 to 88)	Ref.	78 (73 to 83)	Ref.	58 (53 to 64)	Ref.
Less than college	90 (80 to 99)	6 (−5 to 16)	64 (50 to 79)	−14 (−28 to 1)	64 (50 to 77)	5 (−9 to 20)
Qualifying condition						
Cancer pain	76 (65 to 87)	Ref.	66 (53 to 79)	Ref.	52 (39 to 65)	Ref.
Noncancer pain	86 (82 to 90)	10 (−2 to 22)	78 (73 to 83)	12 (−1 to 26)	60 (55 to 66)	8 (−7 to 23)
Pain and interference (PEG) score						
Mild or moderate	81 (75 to 87)	Ref.	76 (69 to 82)	Ref.	56 (48 to 64)	Ref.
Severe	88 (83 to 92)	6 (−1 to 14)	77 (71 to 82)	1 (−8 to 10)	62 (55 to 68)	6 (−4 to 16)
Prescription opioid analgesic use						
No	84 (79 to 89)	Ref.	75 (69 to 81)	Ref.	59 (53 to 65)	Ref.
Yes	86 (80 to 92)	2 (−6 to 10)	78 (71 to 85)	3 (−6 to 12)	59 (51 to 67)	−0.2 (−11 to 10)

Values are regression-adjusted for all characteristics listed in this table.

^a^ Refers to the difference with a10-year change in age.

^*^ *p*<0.05; ^**^p<0.01.

Among those willing to participate, 85% (95% CI 81–89%) wanted to participate because they felt that the research might help other people, and 79% (95% CI 74–83%) felt that doctors need better scientific information about medical cannabis (Table 5). Of respondents not willing to participate, 28% (95% CI 14–42%) reported that the study would take too much of their time, and 28% (95% CI 13–42%) reported that they do not want to provide medical information to researchers.

**Table 5.** **Reasons for Being Willing, or Not Willing, to Participate in Research Among New York State Medical Cannabis Patients with Severe or Chronic Pain (*N*=405)**

	% (95% CI)
Reasons for being willing to participate among those willing	
Research might help other people	85 (81–89)
Doctors need better scientific information about medical cannabis	79 (74–83)
Research might help me	76 (71–81)
I want to contribute to scientific research	62 (56–67)
I might get money for participating	28 (23–33)
My doctor would want me to participate	16 (12–20)
My family or friends would want me to participate	15 (11–19)
Reasons for not being willing to participate among those not willing	
The study would take too much of my time	28 (14–42)
I don't want to provide my medical information to researchers	28 (13–42)
I don't want to provide samples (urine or blood) for lab tests	27 (13–41)
I don't want researchers to ask me personal questions	21 (8–34)
I want more information about the study	20 (7–32)
I don't trust researchers to protect my privacy	18 (6–31)
There is not enough money compensation for my time	16 (5–28)
I want more information about who the researchers are	15 (4–26)
The study won't help me directly	11 (1–21)
I don't want researchers to know I take medical cannabis	10 (0.4–20)
The study won't help anyone	0 (0)

## Discussion

While patient groups, clinicians, and policymakers have called for more research on medical cannabis, to our knowledge, this is the first study that has examined willingness of people with chronic pain to participate in such research. Not only did virtually all respondents support the proposed study but also a vast majority reported that they would enroll if asked. However, with an estimated survey response rate of 30%, willingness to participate in the study described may be as low as 26% (85%×30%) of the broader population. Our findings suggest that long-term studies involving repeated, frequent data collection are of interest to the target population and recruitment would be feasible.

Our findings also suggest that future studies can successfully recruit patients who take opioid analgesics, a subgroup of particular clinical importance. Such patients can have ambivalence about long-term use of opioid analgesics,^47–49^ want research studies to include decreasing opioid dose as an end-point,^50^ and may seek medical cannabis as an alternative or adjunctive therapy.^51^ As suggested by surveys and anecdotal evidence, patients may also seek medical cannabis for the purposes of tapering or discontinuing opioid analgesics.^52–59^ State-level ecological studies have found associations between medical cannabis laws and lower rates of opioid prescribing and overdoses involving opioids.^60–62^ In the era of the opioid epidemic, rigorous patient-level studies of the impact of medical cannabis use on opioid analgesic use are urgently needed.

While patient populations and study designs are distinct, our finding of lower willingness to participate with increasing age is consistent with some, but not all, recent surveys about observational (e.g., biobanking and genomics) and interventional (e.g., clinical trial) research.^19,20,23,28,30,45,63^ The difference by age that we found may be due, at least in part, to the mode of data collection (phone-based), but previous surveys about technology-based studies have found mixed associations between age and willingness to participate.^31,32,46^ Older respondents may also have differing attitudes about research (e.g., concern for experimentation) or face other potential barriers.^23^ To ensure equal representation, future studies of chronic pain and medical cannabis should specifically seek to engage older adults.

While we designed our study description based on previously described information needs of potential study participants with chronic pain,^17,18^ there are several factors which could improve willingness to participate even further. About one third of respondents who were not willing to participate reported wanting to know more information on the study. This may include more information about access to clinicians during the study, more details about the time burden of the study, more information about management of increased pain or adverse events, or even details such as the names and credentials of the investigators.^17^ Willingness to participate may also increase with the offer of personalized feedback at the end of the study (e.g., a report on pain levels and cannabis use), a feature that other potential research participants have reported valuing.^19^

This study has several limitations. First, as we recruited a convenience sample, respondents' views may not be representative of the broader population with chronic pain who take medical cannabis. We could not assess demographic differences between survey respondents and the broader New York medical cannabis patient population because New York has not released these data. Furthermore, as New York's medical cannabis program only distributes extracts that are much more expensive than plant material, eligible patients in New York may have different sociodemographic characteristics than potentially eligible patients in other states. Second, we did not have exact numbers of eligible patients, and our estimated number (and response rate) may not be accurate. Third, we asked about willingness to participate in a hypothetical study, responses to invitations to enroll in an actual study may be different. Finally, we relied on self-report for cannabis and opioid analgesic use and did not confirm with medical records.

## Conclusion

In conclusion, we found high levels of support for a longitudinal cohort study with frequent data collection among patients with chronic pain who take medical cannabis. To the extent that future studies can reach a broad selection of patients, our findings also suggest that recruitment of representative samples is possible. While access to medical cannabis has expanded greatly, interventional research on the efficacy of cannabis for pain is still limited due to funding and regulations. Strong observational studies have the potential to fill in evidence gaps, and the current study demonstrates that patients are willing to participate.

## Supplementary Material

Supplemental data

## Abbreviations Used

CBD: cannabidiol
RCTs: randomized controlled trials
THC: tetrahydrocannabinol
