# Supplementary material for: Willingness to Participate in Longitudinal Research Among People with Chronic Pain Who Take Medical Cannabis: A Cross-Sectional Survey
Source: Cannabis Cannabinoid Res. 2018 Mar 1;3(1):45–53. doi: 10.1089/can.2017.0051 (PMC5870058; doi:10.1089/can.2017.0051)
Supplement: Supplemental data [file Supp_Data.pdf]

Supplementary Data

Below is the description of the cohort study presented to survey respondents:

"A university research group is thinking of doing a study with people with chronic pain who are also starting to take medical marijuana. Because marijuana is illegal in much of the United States, doctors do not have much scientific information about the use of medical marijuana to treat pain. The proposed study would help doctors understand how medical marijuana affects pain and the use of other pain medications over time.

Before we decide whether to do the study or not, we want to know what people think about the idea.

The study would collect information (like pain levels and medication use) and lab tests (like urine and blood tests) over time from volunteers with chronic pain who are starting to take medical marijuana. The study would NOT involve any experimental medications or treatments. Information and samples collected in the study would NOT ever be sold or used to make money. Data from the study would only be used to help patients with chronic pain and their doctors.

Before a person decided to take part, someone from the study would explain the risks and benefits of participating. All participants would have time to ask questions and discuss any concerns they had. All participants would give their informed consent to be in the study.

Researchers would follow participants' pain over time. Participants would give the study access to all of their medical records, including information about their pain and medication use. They would have a short visit with a study doctor every 3 months or more to answer surveys, assess physical functioning (walking down a hallway, getting up out of a chair, bending over, and touching toes), and do lab tests (urine and blood). Between visits, participants might be asked to do other things like answer surveys or track their medication use with a cell phone or smartphone. For all surveys and activities, participants would be compensated for their time with a small amount of cash or a gift card: about $50 for in-person visits and $5 for brief 5-minute cell phone surveys. Over the course of the year, participants could get about $600.

The samples, information, and lab test results would all be 'coded'. 'Coded' means that the names and personal information of each participant would be replaced with a number. The names and personal information of each participant would then be removed. All coded information would be stored in a secure database and only the researchers would have access to it."
